# Cannabidiol Regulates Long Term Potentiation Following Status Epilepticus: Mediation by Calcium Stores and Serotonin

**DOI:** 10.3389/fnmol.2018.00032

**Published:** 2018-02-06

**Authors:** Nicola Maggio, Efrat Shavit Stein, Menahem Segal

**Affiliations:** ^1^Department of Neurology, The Chaim Sheba Medical Center, Ramat Gan, Israel; ^2^Department of Neurology and Neurosurgery, Sackler Faculty of Medicine, Tel Aviv University, Tel Aviv, Israel; ^3^The Sagol School of Neuroscience, Tel Aviv University, Tel Aviv, Israel; ^4^Department of Neurobiology, The Weizmann Institute of Science, Rehovot, Israel

**Keywords:** status epilepticus, synaptic plasticity, LTP, cannabidiol, serotonin, hippocampus

## Abstract

Epilepsy is a devastating disease, with cognitive and emotional consequences that are not curable. In recent years, it became apparent that cannabinoids help patients to cope with epilepsy. We have studied the effects of cannabidiol (CBD) on the ability to produce long term potentiation (LTP) in stratum radiatum of CA1 region of the mouse hippocampus. Exposure to seizure-producing pilocarpine reduced the ability to generate LTP in the slice. Pre-exposure to CBD prevented this effect of pilocarpine. Furthermore, CBD caused a marked increase in ability to generate LTP, an effect that was blocked by calcium store antagonists as well as by a reduction in serotonin tone. Serotonin, possibly acting at a 5HT1A receptor, or fenfluramine (FFA), which causes release of serotonin from its native terminals, mimicked the effect of CBD. It is proposed that CBD enhances non-NMDA LTP in the slice by facilitating release of serotonin from terminals, consequently ameliorating the detrimental effects of pilocarpine.

## Introduction

Epilepsy is a chronic neurological disease, most likely caused by a long-lasting shift in excitation/inhibition balance in specific vulnerable regions in the brain. The causes for the emergence of the disease in most cases are not known, except for head trauma, juvenile febrile seizures and genetic predisposition to epilepsy (Roopra et al., 2012; Cendes et al., 2014; Kobow and Blümcke, 2014; Fiest et al., 2017; Politsky, 2017). Furthermore, a large fraction of epileptic patients is refractory to medication, resulting in a marked deterioration of the patient’s quality of life. Animal models of epilepsy employ methods aimed at increasing excitation (e.g., kainic acid), or blockade of synaptic inhibition (e.g., bicuculline). Several methods are used to slow down/stop epileptic seizures in experimental animals, with the most effective one involving an increase in GABAergic tone (Wahab et al., 2010; Sebe and Baraban, 2011; Ferando and Mody, 2012). The onset, duration and intensity of an epileptic seizure in an animal model of epilepsy can be affected by environmental, sensory or affective parameters. In earlier studies, we described a bi-directional interaction between stress experienced either before or after exposure to pilocarpine (used to trigger an epileptic seizure) on the development of epilepsy (Maggio et al., 2017), indicating that neuromodulation can alter reactivity to epileptogenic stimulation in a complex manner.

In recent years, there is increasing confirmation and extension of earlier anecdotal evidence for the regulation of epileptic seizures by cannabinoids (Katona, 2015; Hosseinzadeh et al., 2016). Cannabis sativa (marihuana) contains several active compounds that exert diverse actions in the brain (Abush and Akirav, 2010; Korem et al., 2016). One of which is the psychoactive compound ∆^9^THC, acting at a CB1 receptor (Lupica et al., 2017). Activation of CB1 receptors was found to impair long term potentiation (30–60 min, LTP) in the hippocampus, and was assumed to involve activation of several downstream mechanisms (Navakkode and Korte, 2014). The second most abundant cannabinoid is cannabidiol (CBD), a non-psychotropic compound that acts at a non-CB1 receptor, to induce anxiolytic, antidepressant and anti-epileptic action in animals (Jones et al., 2009). The mechanism through which CBD exerts its diverse action remains controversial. In one study (Mao et al., 2015) chronic administration of CBD was found to cause a reduction in NR1 receptors in the hippocampus, leading to a decrease in ability to undergo synaptic plasticity. In contrast, it was reported that CBD enhances GABA and serotonin (5HT) release (Drysdale et al., 2006; Zanelati et al., 2010; Kaplan et al., 2017) which may enhance excitability of the tissue. In the present study we focused on the ability of CBD to counteract the chronic effect of pilocarpine on expression of LTP in CA1 region of the mouse hippocampus, and explored the possibility that this action is exerted through release of calcium from intracellular stores and activation of the 5HT1 receptor.

## Materials and Methods

### Animal Handlings and Seizure Induction

Animal handling was approved by the Institutional Animal Care and Use Committee at the Chaim Sheba Medical Center, which adheres to the national law and NIH rules (protocol number: #1022/16). Four/five-month-old male C57BL/6J mice were used throughout the study. Status Epilepticus (SE) was induced by intraperitoneal (i.p.) injection of 250 mg/kg pilocarpine (Sigma Aldrich, pilocarpine hydrochloride). In order to avoid side effects induced by peripheral cholinergic activation, mice were treated with atropine methyl nitrate (AMN, 1 mg/kg, i.p.; Sigma Aldrich), a muscarinic acetylcholine receptor antagonist which does not cross the blood brain barrier 30 min before pilocarpine injection, while diazepam (3 mg/kg, i.p.) was used to halt convulsions prior to experimental assessment. Control groups received the same treatment with atropine and diazepam, however pilocarpine was not injected. After pilocarpine injection, behavioral seizure activity was documented every 10 min by an investigator blind to experimental conditions using the modified Racine’s stages as previously described (Lenz et al., 2017; c.f. 0 = no seizures, 1 = freezing, 2 = single twitches, 3 = orofacial seizures, 4 = clonic seizures, 5 = tonic seizures, and 6 = death). CBD (Sigma Aldrich, Rehovot Israel) was injected i.p. at a dose of 30 mg/kg as previously published (Izquierdo et al., 1973; Mao et al., 2015) 45 min prior to seizure induction.

### Electrophysiology

Animals were used for the recording experiments 24 h after pilocarpine injections. They were rapidly decapitated and 350 μm coronal dorsal hippocampal slices were used. Slices were incubated for 1.5 h in a humidified, carbogenated (5% CO_2_ and 95% O_2_) gas atmosphere at 33 ± 1°C and were perfused with artificial CSF [containing (in mM) 124 NaCl, 2 KCl, 26 NaHCO_3_, 1.24KH_2_PO_4_, 2.5 CaCl_2_, 2MgSO_4_ and 10 glucose, pH 7.4] in a standard interface chamber. In order to avoid hyper excitability, a cut between CA3 and CA1 was made before the experiments. Recordings were made with a glass pipette containing 0.75 M NaCl (4 MΩ) placed in the stratum radiatum CA1. Stimulation was evoked using a Master 8 pulse stimulator (A.M.P.I., Jerusalem, Israel) and was delivered through two sets of bipolar nichrome electrodes placed on either side of the recording electrode to probe two independent pathways in the same slice as previously described (Maggio and Segal, 2007a). LTP was induced by high-frequency stimulation consisting of 100 pulses at twice the test intensity, delivered at a frequency of 100 Hz (HFS; 100 Hz, 1 s). Before and after the tetanic stimulation, stimulation was applied at a frequency of 0.033 Hz. Responses were digitized at 5 kHz and stored on a computer. Off-line analysis and data acquisition were performed using Spike 2 software (CED, Cambridge, England). All numerical data are expressed as mean ± SEM, and EPSP slope changes after tetanic stimulation were calculated with respect to baseline. There were no systematic differences in the magnitudes of the baseline responses in the different conditions. All values reported refer to 30 min after tetanic stimulation.

### Statistics

Each experimental group consisted of 12 slices taken from four mice. Where appropriate, statistical analysis was performed with analysis of variance (ANOVA) followed by *post hoc* Tukey’s comparisons.

## Results

**Figure 1:**
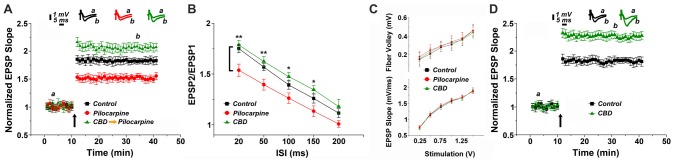
Cannabidiol (CBD) restores long term potentiation (LTP) in pilocarpine treated animals. **(A)** LTP tested 24 h following status epilepticus (SE). A systemic injection of 30 mg/kg CBD rescues LTP in pilocarpine treated animals. **(B)** There was a reduction in paired pulse potentiation by pilocarpine, but a marginal facilitating effect of CBD, at all paired pulse intervals (*p < 0.05, **p < 0.01). **(C)** No difference in input/output relations, measured either in the fiber volley (top) or EPSP slopes (bottom). **(D)** CBD applied to naïve mice also caused an increase in LTP production. Averaged EPSP are plotted vs. time. Representative traces at indicated times (a,b) are shown on top of each section, *n* = 12 slices for each experiment, refer to text for statistics.

In the first series of experiments we replicated our earlier observations on the long term effects of pilocarpine on the ability to induce LTP of reactivity of CA1 neurons to stimulation of stratum radiatum of slices of the dorsal hippocampus of the mice (Maggio et al., 2017). We now examined whether CBD can rescue LTP in these conditions. There was no difference among the groups in input/output relations (Figure 1C), indicating that basic synaptic transmission has not been altered by the epileptic treatment. However, there was a marked reduction in ability to induce LTP in the pilocarpine treated mice (Figure 1A), as seen before (Maggio et al., 2017). This difference was apparent already just after the tetanic stimulation (1.85 ± 0.07 in control vs. 1.53 ± 0.06 in pilocarpine treated animals respectively), indicating that the initial trigger for LTP is significantly reduced at 24 h after the pilocarpine treatment. The reduction in the magnitude of LTP was accompanied by a reduction in paired pulse potentiation (specifically 1.78 ± 0.04 and 1.62 ± 0.05 for controls; 1.53 ± 0.06 and 1.39 ± 0.05 for pilocarpine; 1.74 ± 0.04 and 1.56 ± 0.03 for CBD at ISIs of 20 ms and 50 ms respectively, Figure 1B), in congruence with initial reduction in LTP, indicating that short term potentiation is also impaired by pilocarpine. A 2-ways ANOVA analysis comparing different treatments (Factor A) and different ISIs intervals (Factor B) revealed a significant difference for Factor A (*F* = 25.8; *p* < 0.001; *n* = 12 slices for each group) and for Factor B (*F* = 68.83; *p* < 0.001; *n* = 12 slices for each group) however no significant difference was detected at the interaction between them (*F* = 0.21; *p* = 0.98; *n* = 12 slices for each group). A *post hoc* Tuckey comparison revealed major statistical significant difference between the pilocarpine and CBD groups at 20 ms and 50 ms (*p* < 0.001; *n* = 12). In sharp contrast, pretreatment with CBD before the injection of pilocarpine, not only abrogated the effect of the later drug, but in fact enhanced the magnitude of LTP significantly above control levels (specifically at the 34th minute of recording: 1.81 ± 0.04, 1.54 ± 0.055 and 2.08 ± 0.07 for control, pilocarpine and CBD-pilocarpine respectively, *n* = 12 slices for each group; Figure 1A). A 1-way ANOVA comparing the different treatment groups revealed a significant difference among them (*F* = 95.12; *p* < 0.001) with a *post hoc* Tuckey comparison revealing the highest significant difference between the pilocarpine and CBD-pilocarpine groups (*p* < 0.0001). Furthermore, CBD injected *in vivo* 24 h before the sacrifice of the mouse for the slice experiment enhanced production of LTP significantly above control levels (at the 34th minute of recording: 1.83 ± 0.05 for control vs. 2.23 ± 0.06 for CBD respectively, *p* < 0.001; *n* = 12 slices for each group; Figure 1D).

The possibility that CBD affects directly the animal reactivity to pilocarpine through some central mechanism was examined by observing behavior of the mice after the injection of the latter drug (Supplementary Figure S1). When pilocarpine was preceded by an i.p. injection of CBD, the behavioral symptoms were milder than those seen after injection of pilocarpine alone as suggested before (Izquierdo et al., 1973). A 2 ways ANOVA comparing different treatments (Factor A) and different time points (Factor B) revealed a significant difference for Factor A (*F* = 4.17; *p* < 0.04; *n* = 30 animals for each group) and for Factor B (*F* = 6.3; *p* < 0.001; *n* = 30 animals for each group) however no significant difference was detected at the interaction between them (*F* = 0.34; *p* = 0.94; *n* = 30 animals for each group). A *post hoc* Tuckey comparison revealed major statistical significance among the two groups at 90 min (*p* < 0.05; *n* = 30 animals for each group; Supplementary Figure S1).

**Figure 2:**
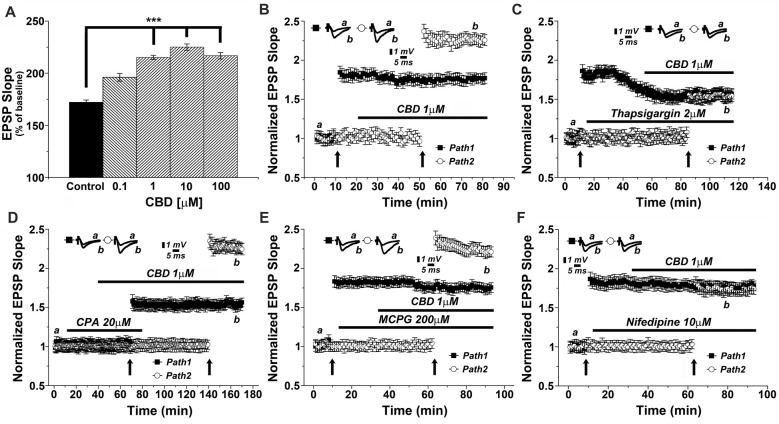
CBD enhances LTP through release of calcium from stores, and through voltage-gated calcium channels (VGCCs) dependent mechanisms. **(A)** CBD applied at concentration of 0.1, 1, 10 and 100 μM enhances LTP in hippocampal slices (***p < 0.001). **(B)** In a two pathways protocol, used in this and all following figures, application of 1 μM CBD after a tetanic stimulation of one pathway enhanced LTP in response to the other pathway, without an effect on the already tetanized pathway. **(C)** The non-competitive inhibitor of the sarco/endoplasmatic reticulum Ca^2+^ ATPase (SERCA) pump Thapsigargin (2 μM) applied to the slice reduced the already tetanized pathway, and blocked the CBD mediated effect on LTP. **(D)** The reversible inhibitor of SERCA pumps cyclopiazonic acid (CPA, 20 μM) transiently blocked the effects of CBD on LTP. Upon washout of CPA, the effect of CBD (1 μM) on LTP was restored. **(E)** The metabotropic glutamate receptor antagonist MCPG (200 μM) was unable to block the effects of CBD on LTP. **(F)** The L-type VGCCs antagonist nifedipine (10 μM) blocked the effects of CBD on LTP. Representative traces at indicated times (a,b) are shown on top of each section, *n* = 12 slices for each experiment (see text for further details).

We then conducted a series of experiments, in an attempt to analyze the molecular basis of CBD action in the slice. This was done with the drug perfused into the slice, and the two pathways stimulation protocol was employed to examine the effects of CBD in presence of different drugs (Figure 2). Initially, we examined the concentration-dependent effect of CBD on tetanic stimulation induction of an increase in LTP slope (Figure 2A). For further experiments we selected a concentration of 1 μM, which produced a highly significant and consistent rise in EPSP slopes following tetanic stimulation. It should be added that at this concentration, CBD did not have any noticeable effect on basal synaptic properties recorded in the slice (Figure 2B). In presence of CBD, perfused into the slice for 30 min, LTP induced by a tetanic stimulation amounted to 2.27 ± 0.07 above baseline, well above control level of LTP (1.73 ± 0.06; *p* < 0.001; *n* = 12 slices) induced by prior stimulation of the other pathway in the two pathway protocol (Figure 2B). It should be noted that CBD did not affect the response to the first pathway, where LTP was already established (ibid).

As it has been proposed that CBD causes release of calcium from intracellular stores (Drysdale et al., 2006) we examined the effects of several drugs known to interact with neuronal calcium stores on reactivity to CBD. Thapsigargin (2 μM), which blocks the endoplasmic reticulum calcium pump irreversibly, was applied after a tetanic stimulation to one of two pathways, following the establishment of LTP in this pathway, and before exposure to CBD and the tetanic stimulation to the other pathway (Figure 2C). Strikingly, while thapsigargin did not affect basal synaptic responses, it did reduce markedly the already potentiated response (1.58 ± 0.05 vs. 1.86 ± 0.08 when potentiation was established at pathway 1), as previously observed (Maggio and Segal, 2007b). Furthermore, following exposure to CBD, the tetanic stimulation of the second pathway failed to induce a large potentiation, as seen before, and in fact, CBD was totally ineffective in presence of thapsigargin (specifically at the 104th minute of recording: 1.59 ± 0.05 for pathway 1 vs. 1.56 ± 0.07 for pathway 2, respectively; *p* = 0.082, *n* = 12 slices; Figure 2C).

We further explored the role of calcium stores in the effects of CBD, using cyclopiazonic acid (CPA), which is a highly selective and reversible inhibitor of the SERCA (sarcoplasmic/endoplasmic reticulum calcium ATPase) pump. At 20 μM, CPA had no apparent effect on basal EPSPs, but it blocked the effect of CBD on LTP (Figure 2D). Upon extensive washout of CPA for over an hour, CBD effect on LTP was fully restored (specifically at the 156th minute of recording: 1.58 ± 0.06 for pathway 1 vs. 2.28 ± 0.05 for pathway 2; *p* < 0.0001; *n* = 12 slices; Figure 2D).

MCPG, a metabotropic glutamate receptor antagonist, which blocks the rise of IP3 inside neurons, hence the IP3-dependent release of calcium from stores, was tested at a high dose of 200 μM. Once again, MCPG did not affect basal EPSPs, nor the already established LTP in one pathway or the effect of CBD on LTP in the other pathway (specifically at the 84th minute of recording: 1.76 ± 0.06 for pathway 1 vs. 2.28 ± 0.07 for pathway 2; *p* < 0.001, *n* = 12 slices; Figure 2E), indicating that the IP3 pathway is not likely to be involved in the effect of CBD on LTP.

Nifedipine, a voltage-gated calcium channel (VGCC) blocker was tested (at 10 μM concentration) in 12 slices. While it did not affect basal EPSPs or the already established LTP in one channel, it completely blocked the ability of CBD to further enhance LTP in the second channel of the two (specifically at the 84th minute of recording: 1.75 ± 0.07 for pathway 1 vs. 1.70 ± 0.05 for pathway 2; *p* = 0.25, *n* = 12 slices; Figure 2F).

**Figure 3:**
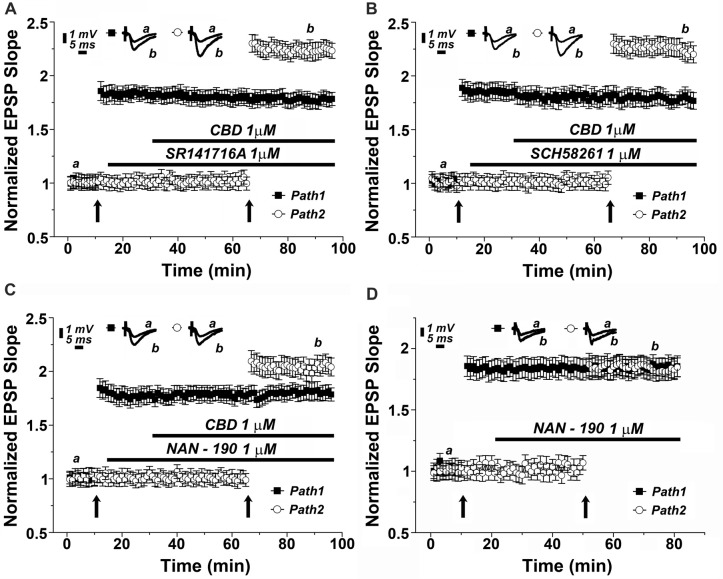
The 5HT1_A_ serotonin receptor mediates the effects of CBD on LTP. Neither the **(A)** CB1 antagonist (SR141716A; 1 μM), nor **(B)** the adenosine A_2A_ antagonist (SCH58261; 1 μM) blocked the effects of CBD on LTP. **(C)** The 5HT-1_A_ antagonist (NAN-190; 1 μM) partially blocked the CBD- enhanced LTP in hippocampal slices. Representative traces at indicated times (a,b) are shown on top of each section, *n* = 12 slices for each experiment. **(D)** The 5HT1A antagonist NAN-190 did not affect LTP produced by a standard tetanic stimulation, when comparing potentiated responses in the two pathways protocol, before and during exposure to NAN-190.

There are two main receptor types for the cannabinoids, CB1, which is the most abundant cannabinoid receptor in the brain, and CB2, which predominates in the periphery, but also in some central structures (Lu and Anderson, 2017; Lupica et al., 2017). To test if the effect of CBD is mediated by a CB1 receptor as suggested elsewhere (Lauckner et al., 2005), we applied a selective CB1 receptor antagonist (SR141716A) at a concentration of 1 μM (Figure 3A). Clearly, the drug did not have an effect of its own, and neither did it affect the ability of CBD to facilitate the reactivity to the tetanic stimulation (specifically at the 87th minute of recording: 1.78 ± 0.06 for pathway 1 vs. 2.21 ± 0.08 for pathway 2, *p* < 0.001, *n* = 12 slices), indicating that CBD is not likely to act through a CB1 receptor.

CBD is suggested to interact with the adenosine A2 receptor, to provide an anti-inflammatory reaction in the brain and periphery (Mecha et al., 2013). We tested the possible blockade of CBD action by the adenosine A2 receptor selective antagonist, SCH58261. At a dose of 1 μM, this drug was totally non-effective (specifically at the 87th minute of recording: 1.76 ± 0.06 for pathway 1 vs. 2.27 ± 0.08 for pathway 2, *p* < 0.001, *n* = 12 slices; Figure 3B).

It has been proposed recently that CBD acts in the brain through activation of a 5-HT1A receptor (Linge et al., 2016). This was examined in another series of slices, which was exposed to a 5HT1A antagonist (NAN-190) at a dose of 1 μM. The drug did not affect basal EPSPs, or already established LTP (Figure 3C), but it markedly reduced the ability of CBD to facilitate the induction of LTP in the second path of the slice (specifically at the 87th minute of recording: 1.82 ± 0.08 for pathway 1 vs. 1.98 ± 0.07 for pathway 2, *p* = 0.075, *n* = 12 slices). This reduction was significant when compared with the effects of CBD alone (as in Figures 2B [*p* < 0.05], 3A,B). This indicates that CBD may at least partly act by an interaction with the 5HT1A receptor. Such an interaction can be exerted either if CBD facilitates the action of 5HT at the synapse, or by facilitating release of 5HT from terminals. To examine the possible effects of NAN-190 on ability to generate LTP, we exposed the slices to the compound before and after induction of LTP in the two pathways protocol. NAN-190 had no effect on either already established LTP or on its induction in the second pathway, which, incidentally, was similar to the induced LTP in the first pathway (Figure 3D).

**Figure 4:**
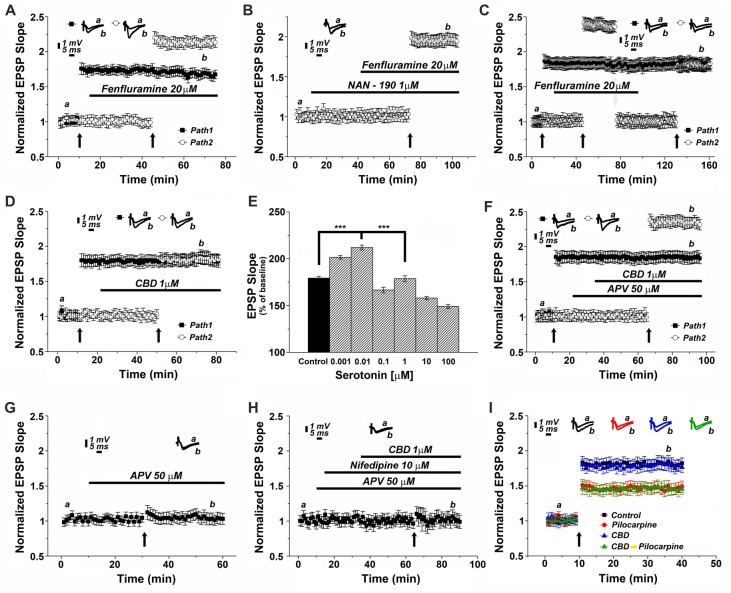
CBD enhances LTP through the release of endogenous serotonin. **(A)** The serotonin releaser fenfluramine (FFA, 20 μM) by itself enhanced LTP, much to the same level as produced in earlier experiments by CBD. **(B)** NAN-190 reduced the effects of FFA on LTP in a one pathway experiment FFA alone caused a potentiation of about 2.20 (as in **A**), however with a wash in of NAN, FFA causes a potentiation of about 1.95. See “Results” section for the statistical analysis. **(C)** FFA facilitates LTP in the second of two pathways, without affecting already established LTP in the first pathway. Following the establishment of the potentiated response, stimulation intensity is reduced manually to the baseline level, FFA is washed out, and the same pathway is tetanized again, to reach same facilitated level as the control pathway, indicating that FFA did not saturate the ability of the tissue to express further potentiation level. **(D)** Serotonin had a biphasic, concentration dependent effect on LTP, facilitating it at low concentration, and depressing LTP at concentrations higher than 0.01 μM. **(E)** CBD (1 μM) was not able to enhance LTP in hippocampal slices that were pre-exposed to FFA for 90 min prior to the experiment, indicating a possible depletion of endogenous 5HT from terminals (***p < 0.001). **(F)** The effect of CBD is not likely to be mediated by activation of the NMDA receptor, as exposure to the NMDAR antagonist APV did not block the facilitating action of CBD on LTP. **(G)** APV alone blocks LTP induced by a tetanic stimulation, in the absence of CBD. **(H)** CBD in presence of both APV and nifedipine is unable to evoke LTP. **(I)** CBD was not able to rescue LTP in slices taken from pilocarpine treated mice, even if CBD was injected to the mice before pilocarpine, when the slices were exposed to FFA before recording commenced.

To test the possibility that CBD causes release of 5HT from terminals and thereby facilitate LTP induction, we first tested the effects of a 5HT releaser, fenfluramine (FFA), on LTP. Acute application of 20 μM FFA (Figure 4A) did not affect basal EPSP, nor did it affect the already potentiated pathway. However, exposure to FFA facilitated the induction of LTP, much the same way as CBD did in previous experiments, and to a similar magnitude (specifically at the 62nd minute of recording: 1.63 ± 0.07 for pathway 1 vs. 2.13 ± 0.05 for pathway 2, *p* < 0.001, *n* = 12 slices). To verify that FFA acts by release of serotonin, it was applied in the presence of NAN-190. Under these conditions, the effect of FFA on LTP was markedly attenuated (at the 98th minute of recording: 1.96 ± 0.07, *n* = 12 slices; Figure 4B; *p* < 0.05 when compared to FFA alone Figure 4A). To examine if FFA saturates the ability to generate LTP, it was first applied after tetanization of one pathway (Figure 4C), resulting in LTP, and before tetanization of the second pathway, which expressed the enhanced LTP, as seen before (Figure 4A). The drug was washed out, the stimulation intensity was reduced to produce a baseline level, and in the absence of the drug, the pathway was tetanized again, to reach the level of potentiation, seen in the unexposed pathway (specifically at the 154th minute of recording: 1.81 ± 0.08 for pathway 1 vs. 1.78 ± 0.06 for pathway 2, *p* = 0.57; *n* = 12 slices). This indicates that FFA does not saturate the ability to generate LTP in the same pathway. Strikingly, continuous presence of FFA throughout the incubation period prior to recording causes an eventual depletion of serotonin (Rothman et al., 2003). Under these conditions, CBD was no longer able to facilitate induction of LTP (specifically at the 72nd minute of recording: 1.85 ± 0.095 for pathway 1 vs. 1.83 ± 0.08 for pathway 2; *p* = 0.80; *n* = 12 slices; Figure 4D).

Since serotonin has been suggested to exert opposing effects on ability to generate LTP (Staubli and Otaky, 1994; Shakesby et al., 2002), we exposed slices to different concentrations of serotonin (Figure 4E), to find a bi-direction effect on magnitude of LTP, a facilitating action at very low concentrations (0.001 and 0.01 μM) and a suppressive action at higher concentrations of serotonin, as seen elsewhere (ibid). This may be explained by serotonin acting at two separate receptors, a high and low affinity one, or by its action of different neuronal compartments or neuron types (Segal, 1990), and further experiments will have to clarify these possibilities.

To examine if CBD facilitates LTP by interacting with the NMDA receptor, its antagonist, 2APV (at 50 μM) was washed in before exposure to CBD. Under these conditions, CBD was still able to facilitate induction of LTP, indicating that it is not mediated by an interaction with the NMDA receptor (specifically at the 87th minute of recordings: 1.82 ± 0.08 for pathway 1 vs. 2.33 ± 0.08 for pathway 2, *p* < 0.001; *n* = 12 slices; Figure 4F). Indeed, APV alone blocked LTP (Figure 4G), thus further supporting the NMDA-independence of CBD evoked LTP. Finally, in yet another series of experiments we applied CBD in presence of both APV and nifedipine (Figure 4H). In this setting, LTP was totally blocked, suggesting that CBD-induced LTP is indeed mediated by the activation of VGCCs.

We then reverted to the experiments presented in Figure 1, to examine if the antagonistic action of CBD on the effect of pilocarpine injected 24 h earlier on LTP is mediated by serotonin. Indeed, exposure of the slices before recording to FFA for 90 min blocked the action of CBD on the ability to induce LTP (A 1-way ANOVA analysis aimed at comparing the different groups revealed a significant difference among them; *F* = 24.74, *p* < 0.0001; *n* = 12 slices for each group; Figure 4I), indicating that this action of CBD is likely to be mediated by release of serotonin from terminals within the hippocampus.

## Discussion

The present results illustrate a striking effect of CBD on the ability to generate LTP in CA1 region of mouse hippocampal slices. The drug counteracts the effect of seizure-producing pilocarpine on LTP generation, is facilitating the generation of LTP on its own, and is likely to be mediated by calcium stores in neurons and possibly glia in the slice. The effect of CBD is likely to be mediated by release of serotonin from terminals in the hippocampus, as these effects are mimicked by acute release of 5HT from terminals by FFA, and are blocked by depletion of serotonin with FFA, and partly by the 5HT1a antagonist NAN190. These results indicate that CBD causes release of serotonin from terminals, which will possibly act on 5HT1A receptors, leading to a functional effect on LTP. These results confirm and extend earlier observations on the effect of CBD on epilepsy and its interaction with the serotonergic system.

Two main types of cannabinoid receptors exist in the brain, the more abundant one CB1R, has been studied extensively, it is likely to involve activation of phospholipase C, which eventually causes an increase in cytosolic calcium by releasing it from stores (Lauckner et al., 2005). In their hands, this effect is not shared with CBD or HU210, other cannabinoid agonists who lack psychoactive properties. Nonetheless, the effects of CBD are strikingly attenuated by thapsigargin and CPA, indicating that CBD effects are also mediated by release of calcium from stores. It is not likely an effect of activation of a CB1R, as the CB1R antagonist does not affect the action of CBD (Figure 3).

Pilocarpine, a cholinergic drug that produces SE in rodents, has been shown by us and others (Maggio et al., 2017) to suppress the ability to generate LTP in hippocampal slices. This is likely to reflect either a reduction in density of afferent fibers, or a homeostatic mechanism which will reduce the number of NMDA receptors in the postsynaptic neurons, to counteract the enhanced excitability. The latter is the more likely possibility, as we observed a marked change in the initial phase following the tetanic stimulation, indicating that our treatments caused an immediate change in ability to recruit NMDA receptors, needed to elevate ambient [Ca^2+^]_i_ and start the plasticity cascade. In support of this assertion is our observation that CBD does not affect basal neurotransmission in the stimulated pathways, indicating that it does not affect AMPA receptors that are activated in the standard recording conditions.

CBD has been shown to counteract epileptic seizures generated in the hippocampus by direct injection of pilocarpine (Do Val-da Silva et al., 2017), and in a mouse model of Dravet syndrome, which involves epileptic seizures. While the mechanisms underlying the effects of CBD have not been explored, it has a profound effect on both epilepsy and associated behavior.

The association of the CBD action with release of 5HT from terminals, and its possible consequent activation of 5HT1A receptors are interesting indeed. Activation of the 5HT1A receptor has been reported to affect LTP in the dentate gyrus of the freely moving rat in a complex, context dependent manner (Sanberg et al., 2006). While NMDA receptors seem not to be involved in the CBD-induced LTP, our observations suggest that the effects of CBD on LTP are mediated through activation of VGCCs. It is not clear at the present time if nifedipine blocks presynaptic or postsynaptic VGCC or both, and the two possible locations may be associated with induction of LTP in some hippocampal pathways (e.g., Lauri et al., 2003). Since nifedipine blocks influx of calcium through VGCC’s but there is no effect of the drug on basal neurotransmission of the excitatory Shaffer collateral pathway, we believe that it affects serotonin-containing axonal terminals and thus hampers the release of serotonin. The mechanisms underlying the selectivity of CBD action on 5HT terminals are still to be investigated.

The effects of serotonin on synaptic activity and in particular on ability to generate LTP has been studied for some time, both *in vivo* and in hippocampal slices (Staubli and Otaky, 1994; Stewart and Reid, 2000; Shakesby et al., 2002; Kojima et al., 2003; Tachibana et al., 2004; Sanberg et al., 2006). Conflicting results, suggesting facilitation or depression of LTP may result from different concentrations of applied and intrinsically released serotonin, and we have replicated this by showing that serotonin at very low concentration facilitates, whereas higher concentrations, as high as those used elsewhere, can suppress LTP. Another difference may result from different preparations, *in vivo* vs. *in vitro*, CA1 vs. dentate gyrus. These differences just illustrate the heterogeneity and complexity of neuromodulation.

It is interesting to speculate about the possible clinical relevance of our results. It has been recognized that there are multiple comorbid conditions associated with epilepsy spanning from anxiety/mood disorders (Kanner, 2011; Brandt and Mula, 2016; Elger et al., 2016) to memory impairments and dementia (Subota et al., 2017). Recent clinical studies have demonstrated a possible anti-epileptic role for CBD in some epilepsy syndromes (Devinsky et al., 2016, 2017; O’Connell et al., 2017), however no studies have so far addressed a putative role for CBD in improving the symptoms of epilepsy comorbidities. In this respect, if CBD restores LTP in epileptic animals, may it improve memory functions in epilepsy patients as well? Besides, if CBD facilitates the release of serotonin in the brain (Zanelati et al., 2010), may it exert anxiolytic effects in epilepsy patients and possibly affect mood disorders in this population? Certainly, clinical data should be collected in order to validate these hypotheses.

All in all, our results link CBD with protection from long term effects of epilepsy on LTP. This is assumed to take place via an effect of CBD on calcium stores which will cause enhanced release of serotonin and facilitate the ability to generate LTP in the hippocampus.

## Author Contributions

NM and MS: conceived the study. NM and ESS: perfomed and analyzed experiments. MS and NM: wrote the article.

## Conflict of Interest Statement

The authors declare that the research was conducted in the absence of any commercial or financial relationships that could be construed as a potential conflict of interest.

## Supplementary Material

FIGURE S1 CBD alters seizure severity in the pilocarpine animal model of Status Epilepticus. Measurements were made in 30 control mice and 30 mice pre-injected with CBD mice. Observations were begun right after i.p injection of pilocarpine. At 90 min after pilocarpine mice were injected with diazepam, to stop convulsions, as detailed elsewhere (Maggio et al., 2017).
