# Supplementary figures and images for: Cannabidiol Regulates Long Term Potentiation Following Status Epilepticus: Mediation by Calcium Stores and Serotonin
Source: Front Mol Neurosci. 2018 Feb 6;11:32. doi: 10.3389/fnmol.2018.00032 (PMC5808210; doi:10.3389/fnmol.2018.00032)

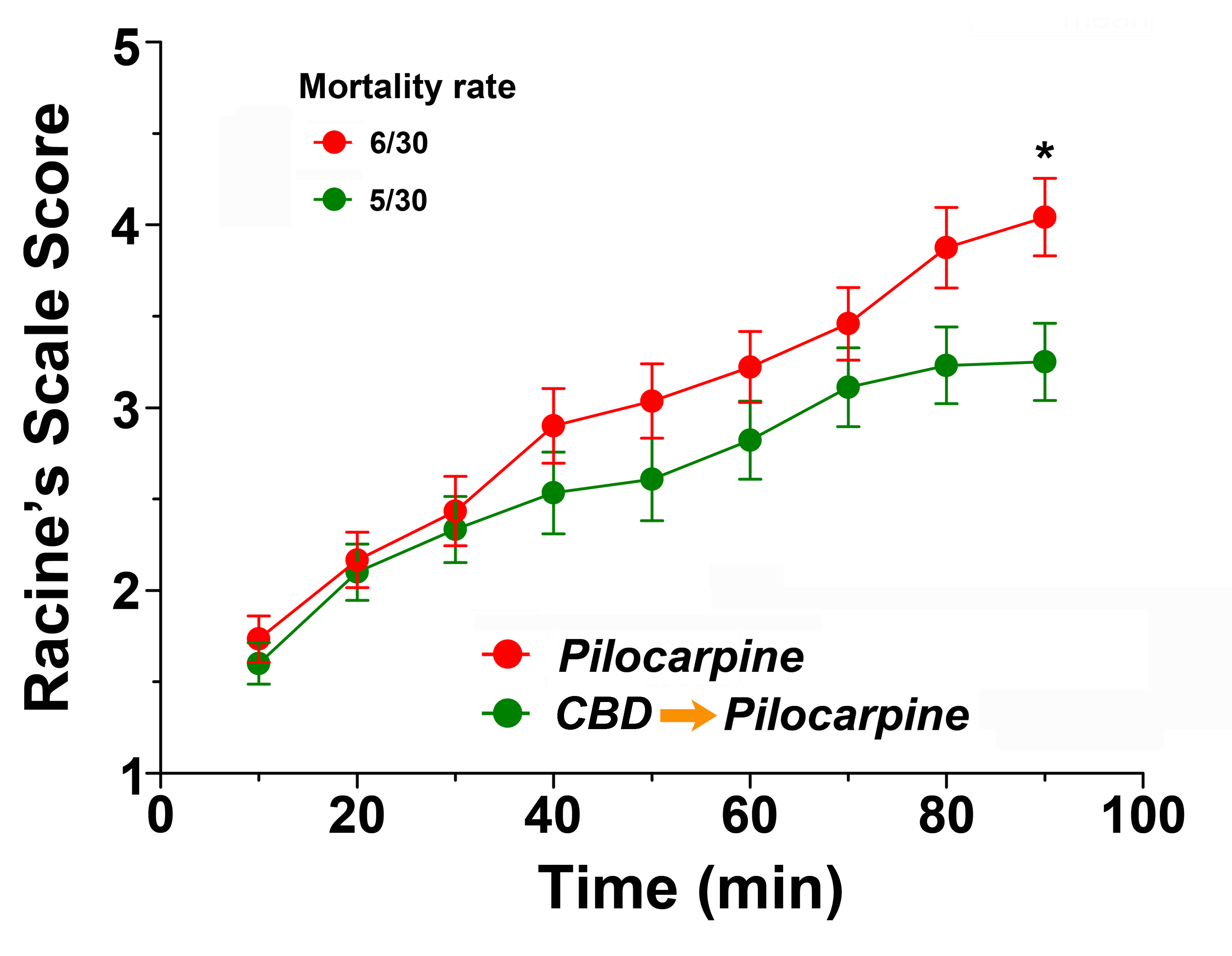

Supplement: FIGURE S1 — CBD alters seizure severity in the pilocarpine animal model of Status Epilepticus. Measurements were made in 30 control mice and 30 mice pre-injected with CBD mice. Observations were begun right after i.p injection of pilocarpine. At 90 min after pilocarpine mice were injected with diazepam, to stop convulsions, as detailed elsewhere (Maggio et al., 2017). [file Image_1.tif]
